# Dual group-based trajectories of physical activity and cognitive function in aged over 55: a nationally representative cohort study

**DOI:** 10.3389/fpubh.2024.1450167

**Published:** 2024-10-29

**Authors:** Xiaotong Wang, Pei Hu, Yating Ai, Shi Zhou, Yucan Li, Pengjun Zhou, Gao Chen, Yuncui Wang, Hui Hu

**Affiliations:** ^1^College of Nursing, Hubei University of Chinese Medicine, Wuhan, China; ^2^Affiliated Hospital of Hubei University of Chinese Medicine, Wuhan, China; ^3^Engineering Research Center of TCM Protection Technology and New Product Development for the Elderly Brain Health, Ministry of Education, Wuhan, China; ^4^Hubei Shizhen Laboratory, Wuhan, China

**Keywords:** physical activity, cognitive function, group-based dual trajectory modeling, older adults, cognitive decline

## Abstract

**Background:**

As individuals age, they commonly experience reduced physical activity and cognitive decline. While evidence, there is limited trajectory research on their concurrent progression and interrelation in individuals over 55 years old.

**Methods:**

The data was collected from 5,765 individuals aged over 55 years who participated in the China Health and Retirement Longitudinal Study (CHARLS) between 2011 and 2020. Physical activity was measured by IPAQ, cognitive function by episodic memory, and mental intactness score. Separate sets of group-based trajectory models were fitted to identify physical activity trajectories and cognitive function trajectories. Multivariate logistic regression was used to estimate the association between baseline characteristics and each set of trajectories. Group-based dual trajectory modeling (GBDTM) was applied to quantify these associations.

**Results:**

GBDTM identified three distinct trajectory groups for physical activity and cognitive outcomes. The physical activity trajectories were classified as “Persistently low physical activity” (74.2%), “Decreasing physical activity” (13.7%), and “Rising physical activity” (12.1%). Similarly, cognitive function trajectories were categorized as “Persistently low cognitive function” (22.2%), “Persistently moderate cognitive function” (37.9%), and “Persistently high cognitive function” (39.9%). Notably, 15.6% of participants followed the trajectories of “Persistently low physical activity” and “Persistently low cognitive function.” The presence of a severe decline in physical activity was associated with an increased likelihood of poor cognitive function and vice versa. Age, sex, education, residential status, BMI, and visual impairment were identified as significant predictors for physical activity and cognitive decline.

**Conclusion:**

This study found that the GBDTM can determine the consistent trajectories of physical activity and cognitive function trajectories that persistently decline in individuals over 55 years. Analyses of predictive factors can be instrumental in promoting physical activity and delaying cognitive decline.

## 1 Introduction

Cognitive impairment is prevalent among older adults, affecting memory, attention, learning, and executive function (1). This is primarily due to the gradual hardening of cerebral blood vessels with age, resulting in cumulative brain damage (2). Initially, individuals experience a gradual decline in their ability to remember, think logically, and perform daily tasks, potentially progressing to severe impairments in daily functioning or dementia in later stages (3). As the global population ages, the prevalence of cognitive impairment will continue to rise (4). Reports suggest that most screening currently focuses on community-dwelling older adults over the age of 60, where the prevalence of mild cognitive impairment is approximately 20%. However, 7.6% of individuals aged 55–59 are still diagnosed with mild cognitive impairment (5), highlighting the importance of early cognitive screening and intervention. Furthermore, as dementia is an irreversible disease with no effective treatment (6), identifying potential risk factors associated with cognitive decline is crucial for slowing the progression of the disease.

The association between physical activity (PA) and cognitive function in older adults has been extensively studied, but the evidence remains inconsistent. For example, Wang et al. (7) analyzed the association between physical activity and memory and executive function at baseline using mixed linear regression methods, while Reas et al. (8) examined the positive impact of regular PA on cognitive function in older adults from a lifespan perspective. Similarly, focusing on the intensity and duration of PA, Wang et al. (9) suggested that moderate-intensity PA at least 6 days per week can slow the decline of cognitive function. Most researchers are optimistic about the role of PA and social activities in slowing cognitive decline. However, some recent high-quality studies suggest that the link between cognitive function and physical activity should be viewed with caution (10). They pointed out that the evidence level of cross-sectional studies is often limited by the inherent design, making it difficult to determine causal relationships or temporal sequences. At the same time, they emphasized the impact of follow-up time, covariates, activity indicators, and cognitive assessment tools on the research outcomes. In addition, most previous longitudinal studies have used linear mixed-effects models (LMMs), which relate repeated measures to random effects and subsequently analyze individual differences in baseline cognitive scores and rates of cognitive decline (11–13). However, physical activities and cognitive function exhibit overall heterogeneity among subjects. Specifically, through individual trajectories across all subjects can vary significantly. Nonetheless, it is also possible that certain individuals share similar developmental trends, thereby forming distinct groups or categories. Kim and Yoon (14) used latent class growth modeling to predict cognitive functioning by analyzing the trajectories of older adults’ participation in social activities. Similarly, Li et al. (15) analyzed cognitive aging trajectories over time using group-based trajectory modeling, suggesting that frequent participation in social and intellectual activities was associated with a reduced risk of cognitive decline and dementia. These studies revealed the heterogeneity of cognitive trajectories and the degree of association between different subgroups, emphasizing the necessity of deeper analysis of the dynamic relationship between PA and cognitive trajectories in older adults.

There are fewer longitudinal studies examining overall activity status and cognition in older adults from a heterogeneous perspective, and it is unclear whether this association varies across subgroups such as age, sex, and depression. Group-Based Dual-Trajectory Models (GBDTM), an extension of Group-Based Trajectory Modeling (GBTM), can effectively analyze the correlation between two measures, exhibiting the probabilistic strength of the association between the two outcomes (16). The aims of our study were (i) to identify the trajectories of PA and cognitive functioning and to describe the baseline characteristics of the physical activity and cognitive functioning trajectory groups, (ii) to estimate the conditional probabilities of different cognitive changes occurring under different physical activity trajectory groups and of different PAs occurring under different cognitive trajectory groups, and (iii) to assess the probability of the associations and the situation of the association between PA and cognition.

## 2 Methods

### 2.1 Background of the study

The China Health and Retirement Longitudinal Study (CHARLS) is a multidisciplinary, cross-sectional social science research project led by the National Development Research Institute at Peking University in collaboration with the China Social Science Survey Center (17). This project systematically collects health, economic status, and retirement arrangements for individuals aged 45 and older, aiming to deeply understand the aging issues in China and provide data support for policy-making. Using a multi-stage stratified random sampling method, the project conducted its first survey among 17,708 participants across 150 counties in 28 provinces during 2011–2012. It has followed up every 2 to 3 years to update data. By 2020, five rounds of data collection were completed (2011, 2013, 2015, 2018, and 2020). The study has been approved by the Biomedical Ethics Committee of Peking University, and all participants have signed informed consent forms (18). Detailed design, sampling procedures, and data collection processes for the CHARLS project can be accessed in prior publications and on the official website.^1^

### 2.2 Study population

This study initially included 13,249 participants aged 55 years and older who participated in the first wave of the survey. A total of 643 were excluded for the following reasons: 297 had been diagnosed with dementia and Parkinson’s disease at baseline; 346 exhibited severe cognitive impairment (overall cognitive scores below 5, which is 1.5 standard deviations below the mean). An additional 6,841 participants were excluded because they were without at least two measurements on PA and cognition from waves 1 to 5. Ultimately, 5,765 participants were included in the longitudinal analyses (see Supplementary Figure S1).

For participants with missing data on variables other than cognitive and PA, the most recent year’s data were used for imputation. If data remained missing, multiple imputation methods were employed (19).

### 2.3 Assessment of physical activity

Combining previous definitions of physical activity (20) and research on Chinese older adults’ activity engagement (21), the evaluation of physical activities employed a standardized self-report questionnaire designed with reference to the International Physical Activity Questionnaire (IPAQ) to classify activity intensity into moderate (22), vigorous, and light intensity. The duration of physical activity in the questionnaire was categorized into five groups: no activity, 10–29 min, 30–119 min, 120–239 min, and 240 min or more. For convenience in calculations, the durations were recoded as 0, 20, 75, 180, and 240 min. By combining the metabolic equivalents (METs) for light, moderate, and vigorous activities with the activity duration (times per week), the total minutes of physical activity were calculated as the sum of these three components (23–25).

Given the variability in scoring methods, to ensure comparability, we analyzed standardized test scores across five waves of data by subtracting the means and dividing by the standard deviations. By subtracting the means and dividing by the standard deviations, we calculated the Z-scores for both physical and social leisure activities (22).

### 2.4 Assessment of cognition

Previous research (7, 26) indicates that cognition assessments primarily focus on episodic memory and mental intactness. Episodic memory was evaluated using a word recall test, including immediate recall (repetition right after reading a 10-word list) and delayed recall (repetition after 10 min), scoring 1 point per word. The cumulative average score ranged from 0 to 20. Mental health (mental state) was evaluated using the TICS battery, which includes tests on serial subtraction, recognizing dates and days, seasons, and simple graph drawing, with a total possible score of 0 to 11. The overall cognitive score, ranging from 0 to 21, is the sum of the episodic memory score and the mental health score. Additionally, z-scores for the cognitive function test data were calculated.

### 2.5 Covariates

In this study, demographic characteristics of participants were collected based on prior literature and clinical practice guidelines (27–30). Assessed variables included age (continuous), sex (male, female), education (no formal education, primary school, middle school, high school, or college and above), marital status (married, other), residency (rural, urban), annual household income (low, medium, high), smoking (never, former, current), drinking (never, former, current), self-reported health (good, fair, poor), number of chronic diseases (0, 1, 2+), and visual hearing impairment (no, yes), and hospitalization (no, yes). Depressive symptoms were assessed using a 10-item version of the Centre for Epidemiological Studies Depression Scale (CES-D), with questions 5 and 8 reverse coded. Each question was scored on a 4-point scale (0–3), with total scores ranging from 0 to 30, indicating increasing severity of depression, with a score of ≥12 indicating the presence of depressive symptoms (29).

The functionality of participants in basic and instrumental activities of daily living was assessed using the ADL (31) and IADL (32) scales. Responses to the first two options indicated normal functioning and were coded as 0, while responses to the last two options indicated a loss of functioning and were coded as 1. Participants were then classified as having 0, 1, or 2+ impairments. Body Mass Index (BMI) was calculated as weight in kilograms divided by height in meters squared and categorized as follows: underweight (<18.5 kg/m^2^), normal (18.5–23.9 kg/m^2^), overweight (24.0–27.9 kg/m^2^), and obese (≥28.0 kg/m^2^) (33).

## 3 Statistical analysis

We used group-based trajectory modeling (GBTM) to identify distinct cognition and activity score trajectories in each wave. GBTM allows for the inclusion of all available scores in the model estimates, assuming that any missing scores occur at random. The successive Z-scores were modeled as censored normal averages (34). A maximum of six trajectory groups was set *a priori* based on the initial analysis. We fitted models ranging from one-group to six-group trajectories. To determine the model with the optimal number of different trajectories, the cubic model was used as a first step and then the model was adjusted according to the *p*-value. We then compared the Bayesian Information Criterion (BIC) to identify the best-fitting model. Furthermore, an average posterior probability (AvePP) of assigning each participant to a group of approximately 70% or higher indicated a good fit. The model is considered highly accurate when its Odds of Correct Classification (OCC) are above 5 for all trajectory groups, and models with more than 10% of participants assigned to each trajectory group were selected (16, 34, 35).

GBDTM allows us to examine the associations between cognitive and physical activity (PA) trajectories through conditional and joint probabilities. Two sets of associative relationships are one-to-one correspondence restricted relationships. Specifically, it includes: (1) the probability of PA trajectories given specific cognitive trajectories, and (2) the probability of cognitive trajectories given specific PA trajectories.

Multivariate logistic regression models were then used to separately estimate the associations between sample characteristics at baseline and the trajectories of PA and cognitive function measures, reporting the odds ratios (ORs) and corresponding 95% confidence intervals (CIs).

### 3.1 Stratified analysis

Finally, the study analyzed stratified analysis by age group (<70 and ≥70 years), sex (male and female), depression (yes, no), functional status (0, 1, ≥2), and analyzed interaction effect between members of the PA and cognitive functioning trajectory group.

All statistical analyses were performed using R packages (http://www.R-project.org, The R Foundation) with PROC TRAJ and Free Statistics software version 1.9.2. All statistical tests were two-sided, and *p* < 0.05 was considered statistically significant.

### 3.2 Sensitivity analysis

First, participants with incomplete baseline data or a diagnosis of dementia, Parkinson’s disease, or severe cognitive impairment were excluded from the comparison of baseline characteristics. Second, participants who were lost to follow-up were compared to those who were ultimately included. Third, studies have shown that trajectory analysis estimation is more stable with three or more measurements; thus, participants with data from three waves were selected to repeat the primary analysis. Additionally, a total of 2,510 participants had cognitive and activity data for all three waves (Supplementary Figures S4, S5).

## 4 Results

### 4.1 Sample characteristics

During the study period from 2011 to 2020, 5,765 participants aged over 55 years old from the China Health and Retirement Longitudinal Study (CHARLS) were included. Of this sample, 45.9% were female, with a mean age at baseline measurement of 64.1 ± 6.9 years. Of these participants, 47.8% had never received any education, 88% were married, 79.4% lived in urban areas, and about 30% were current smokers and drinkers. Nearly half (44.9%) reported multiple chronic diseases, 30.2% rated their health as poor, about 15% reported hearing impairment and visual impairment, and about 25% reported two or more functional impairments.

### 4.2 Cognitive and physical activity trajectory group characteristics

**Figure 1:**
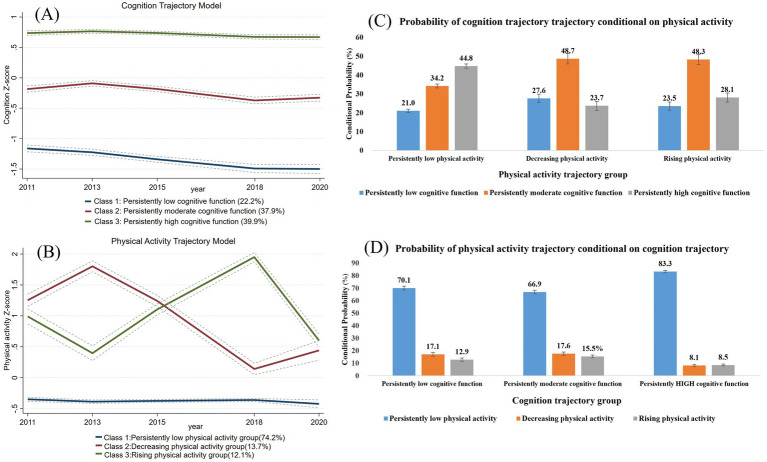
Cognitive function and physical activity trajectories and conditional probabilities linking trajectories of physical activity and cognitive function. The solid line indicates the observed value; the dotted line and the dashed line indicate the predicted value. **(A)** Cognition trajectory model, **(B)** Physical activity trajectory model, **(C)** Probability of cognition trajectory conditional on physical activity, **(D)** Probability of physical activity trajectory conditional on cognitive function.

Groups 2–6 cognitive and physical activity trajectories were fitted to derive the optimal group (Supplementary Tables S1, S2). In the cognitive trajectory group, although the four-group model had the lowest BIC, the mean posterior probability was less than 0.7. Therefore, three trajectories were identified as the optimal model in this study. Figure 1A shows the three trajectory patterns of cognitive functioning: Class 1, “Persistently low cognitive function” (*N* = 1,262, 22.2%), Class 2, “Persistently moderate cognitive function” (*N* = 2,195, 37.9%), and Class 3, “Persistently high cognitive function” (*N* = 2,308, 39.9%).

In the selection of the PA trajectory, since the fourth group accounted for less than 10%, the same three groups were selected as the optimal model. The physical activity data from the 2020 survey may have been influenced by the COVID-19 pandemic that began in 2019. However, the pandemic’s impact on physical activity trends among older adults is complex. While most studies indicate a restriction in physical activity due to the pandemic, some older adults may have prioritized physical activity more as a result of the outbreak (36). Overall, PA also chose the three-group trajectories: Class 1, “Persistently low physical activity “(*N* = 4,349, 74.2%); Class 2, “Decreasing physical activity” (*N* = 722, 13.7%); and Class 3, “Rising physical activity” (*N* = 694, 12.1%). The three cognitive function and physical activity trajectories for the three groups are shown in Figure 1B.

The GBDTM provides a view of the conditional and joint probabilities between physical activity and cognition in a two-trajectory model. Figure 1C illustrates the conditional probabilities of different cognitive decline groups based on varying physical activity trajectories. The percentage of cognitive trajectories in the “Persistently low physical activity” group is essentially similar to the overall percentage of cognitive conditions. Of those in the “Decreasing physical activity” group, 27.6% will experience “Persistently low cognitive function,” and 23.7% will experience “Persistently high cognitive function.” In contrast, in the “Rising physical activity” group, only 23.5% will be in persistently low cognitive function, but 28.1% will be in “Persistently high cognitive function” (Figure 1C). Figure 1D illustrates the conditional probabilities of different physical activity trajectories based on varying cognitive trajectories. Regardless of cognitive trajectory, the most likely physical activity trajectory was “Persistently low physical activity,” with probabilities ranging from 70.1% for “Persistently low cognitive function” to 83.1% for “Persistently high cognitive function.” However, there was a significantly higher proportion of “Decreasing physical activity” (17.1%) than “Increasing physical activity” (12.9%) in the “Persistently low cognitive function.” The probability of persistently low cognitive function was significantly higher than that of “Increasing physical activity” (12.9%), while the opposite was confirmed in the “Persistently high cognitive function” (Figure 1D).

The stratified characteristics are described based on the respective trajectory groups from GBDTM (Tables 1, 2). Table 1 presents the baseline characteristics of overall cognitive functioning for participants in each trajectory group. Participants in the “Persistently low” trajectory group were more likely to be older, have lower levels of education and income, have depressive symptoms, live in a rural area, have more comorbidities, and be current smokers than those in the “Persistently high” trajectory group. Table 2 presents the baseline PA characteristics for participants in each trajectory group. Compared to the “Rising physical activity” trajectory group, participants in the “Decreasing physical activity” trajectory group were more likely to be older, female, have lower education levels and income, and have more comorbidities.

**Table 1:** Baseline characteristics of the participants according to trajectories of cognitive function.

Variables	Class1, Persistently low cognitive function (*N* = 1,262)	Class2, Persistently moderate cognitive function (*N* = 2,195)	Class 3, Persistently high cognitive function (*N* = 2,308)	*p-*value
**Age (years), mean ± SD**	64.1 ± 6.8	62.8 ± 6.2	61.7 ± 5.5	<0.001
**Male, *n* (%)**	695 (55.1)	1,201 (54.7)	1,221 (52.9)	0.34
**Married, *n* (%)**	1,106 (87.6)	1,931 (87.9)	2,034 (88.1)	0.91
**Educational level, *n* (%)**				<0.001
No formal education	710 (56.3)	103 (50.3)	942 (40.8)	
Primary school	308 (24.4)	610 (27.8)	641 (27.8)	
Middle or high school	177 (14.0)	344 (15.7)	448 (19.4)	
College or above	67 (5.3)	138 (6.3)	277 (12.0)	
**Self-report health, *n* (%)**				0.30
Poor	411 (30.3)	681(31.0)	765 (233.1)	
Average	613 (48.6)	1,056 (48.1)	1,047 (45.4)	
Good	238 (17.9)	458 (20.9)	496 (21.5)	
**Depressive symptoms, *n* (%)**	379 (30.0)	597 (27.2)	613 (26.6)	0.08
**Rural residence, *n* (%)**	131 (10.4)	343 (15.6)	714 (30.9)	<0.001
**Comorbidity, *n* (%)**				0.01
0	335 (26.6)	626 (28.5)	570 (24.7)	
1	375 (29.7)	629 (28.7)	641 (27.8)	
≥2	552 (43.7)	940 (42.8)	1,097 (47.5)	
**Annual household income, *n* (%)**				<0.001
Low	454 (35.9)	789 (35.9)	715 (30.9)	
Medium	458 (36.3)	754 (34.4)	694 (30.1)	
High	350 (27.7)	652 (29.7)	899 (39.0)	
**BMI (kg/m**^**2**^**), *n* (%)**				<0.001
Underweight	106 (8.4)	141 (6.4)	149 (6.5)	
Normal	732 (58.0)	1,243 (56.6)	1,141 (49.4)	
Overweight	311 (24.6)	595 (27.1)	772 (33.5)	
Obese	113 (9.0)	216 (9.8)	246 (10.7)	
**Drinking, *n* (%)**				0.11
Never	693 (54.9)	1,202 (54.8)	1,344 (58.2)	
Former	131 (10.4)	249 (11.3)	243 (10.5)	
Current	438 (34.7)	744 (33.9)	721 (31.2)	
**Smoking, *n* (%)**				0.02
Never	708 (56.1)	1,183 (53.9)	1,326 (57.5)	
Former	137 (10.9)	229 (10.4)	286 (12.4)	
Current	417 (33.0)	783 (35.7)	696 (30.2)	
**Visual impairment*, n* (%)**	171 (13.6)	307 (14.0)	402 (17.4)	<0.001
**Hearing impairment, *n* (%)**	194 (15.4)	308 (14.0)	346 (15.0)	0.50
**Hospitalization, *n* (%)**	115 (9.97)	178 (9.04)	169 (8.56)	0.42
**ADL, *n* (%)**				0.07
0	949 (75.2)	1,629 (74.2)	1,683 (73.0)	
1	108 (8.6)	179 (8.2)	169 (7.3)	
≥2	205 (16.2)	387 (17.6)	456 (19.8)	
**IADL, *n* (%)**				0.01
0	916 (72.6)	1,570 (71.5)	1,651 (71.5)	
1	127 (10.1)	218 (9.9)	179 (7.8)	
≥2	219 (17.4)	407 (18.54)	478 (20.7)	

Comorbidity refers to the coexistence of multiple chronic conditions, such as hypertension, diabetes, dyslipidemia, and chronic kidney disease, along with other chronic illnesses diagnosed by physicians, including heart disease, stroke, lung disease, arthritis, and cancer. It is quantified based on the number of these nine chronic diseases present in an individual, categorized as none (0), one (1), or multiple (≥2); Hospitalization refers to whether an individual was hospitalized during the past year.

**Table 2:** Baseline characteristics of the participants according to trajectories of physical activity.

Variables	Class 1, Persistently low physical activity (*N* = 4, 349)	Class2, Decreasing physical activity (*N* = 722)	Class3, Rising physical activity (*N* = 694)	*p-*value
**Age, Mean ± SD**	63.3 ± 6.4	61.5 ± 5.2	60.3 ± 4.5	<0.001
**Male, *n* (%)**	2,309 (53.1)	410 (56.8)	398 (57.4)	0.03
**Married, *n* (%)**	3,827 (88.0)	629 (87.1)	615 (88.6)	0.68
**Educational level, *n* (%)**				<0.001
No formal education	2,005 (46.1)	375 (51.9)	375 (54.0)	
Primary school	1,153 (26.5)	199 (27.6)	207 (29.8)	
Middle or high school	771 (17.7)	121 (16.8)	77 (11.1)	
College or above	420 (9.7)	27 (3.7)	35 (5.0)	
**Self-reported health, *n* (%)**				0.05
Poor	1,340 (31.8)	226 (31.3)	247 (35.6)	
Fair	2,057 (47.3)	344 (47.7)	315 (45.4)	
Good	908 (20.9)	152 (21.0)	132 (19.0)	
**Depressive symptoms, *n* (%)**	1,164 (26.8)	204 (28.3)	221 (31.8)	0.02
**Rural residence, *n* (%)**	1,068 (24.6)	66 (9.1)	54 (7.8)	<0.001
**Comorbidity, *n* (%)**				0.04
0	1,112 (25.6)	215 (29.8)	204 (29.4)	
1	1,255 (28.9)	207 (28.7)	183 (26.4)	
≥2	1,982 (45.6)	300 (41.6)	307 (44.2)	
**Annual household income, *n* (%)**				<0.001
Low	1,443 (33.2)	260 (36.0)	255 (36.7)	
Medium	1,385 (31.9)	266 (36.8)	255 (36.7)	
High	1,521 (35.0)	196 (27.2)	184 (26.5)	
**BMI (kg/m**^**2**^**), *n* (%)**				<0.001
Underweight	271 (6.2)	63 (8.7)	62 (8.9)	
Normal	2,256 (51.9)	440 (60.9)	420 (60.5)	
Overweight	1,344 (30.9)	175 (24.2)	159 (22.9)	
Obese	478 (11.0)	44 (6.1)	53 (7.6)	
**Drinking, *n* (%)**				0.04
Never	2,494 (57.4)	376 (52.1)	369 (53.2)	
Former	457 (10.5)	89 (12.3)	77 (11.1)	
Current	1,398 (32.2)	257 (35.6)	248 (35.7)	
**Smoking, *n* (%)**				<0.001
Never	2,466 (56.7)	377 (52.2)	374 (53.9)	
Former	519 (11.9)	71 (9.8)	62 (8.9)	
Current	1,364 (31.4)	274 (38.0)	258 (37.2)	
**Visual impairment, *n* (%)**	682 (15.7)	87 (12.1)	111 (16.0)	0.04
**Hearing impairment, *n* (%)**	629 (14.5)	113 (15.7)	106 (15.3)	0.64
Hospitalization, *n* (%)	372 (9.8)	38 (5.8)	52 (8.3)	0.003
**ADL, *n* (%)**				0.03
0	3,208 (73.8)	545 (75.5)	508 (73.2)	
1	322 (7.4)	68 (9.4)	66 (9.5)	
≥2	819 (18.8)	109 (15.1)	120 (17.3)	
**IADL, *n* (%)**				0.12
0	3,095 (71.2)	533 (73.8)	509 (73.3)	
1	387 (8.9)	69 (9.6)	68 (9.8)	
≥2	867 (19.9)	120 (16.6)	117 (16.9)	

### 4.3 Association of physical activity and cognition trajectories

Univariate logistic regression analyses for PA and cognition trajectories are presented in Supplementary Tables S7, S8. When using the multivariate logistic model with the identified cognitive function trajectories as the primary independent variable, a significant association with the PA trajectories was observed (Table 3). Age, residency, and educational level were significant predictors for the “Persistently moderate cognitive function” and the “Persistently high cognitive function” groups. For the “Persistently high cognitive function” group, being female may have been a cognitive risk factor (OR = 0.79, 95% CI: 0.68–0.92), whereas a steadier and increased PA was a vital protective factor.

**Table 3:** Multivariate logistic regression analyses with cognitive function trajectory groups.

Variables	Cognitive function trajectories *(ref: Persistently Low cognitive function)*			
	Persistently moderate cognitive function		Persistently high cognitive function	
	OR (95%CI)	*p*-value	OR (95%CI)	*p*-value
**Physical activity (ref: decreasing physical activity)**				
Persistently low physical activity	0.95 (0.78 ~ 1.16)	0.63	2.84 (2.25 ~ 3.59)	<0.001
Rising physical activity	1.14 (0.88 ~ 1.47)	0.32	1.48 (1.09 ~ 2)	0.01
**Age**	0.97 (0.96 ~ 0.98)	<0.001	0.92 (0.91 ~ 0.93)	<0.001
**Female (vs. male)**	0.92 (0.79 ~ 1.06)	0.24	0.79 (0.68 ~ 0.92)	0.003
Marital status (vs. married)	1.02 (0.82 ~ 1.27)	0.85	1.00 (0.80 ~ 1.26)	0.99
**Educational level (vs. no formal education)**				
Primary school	1.29 (1.08 ~ 1.53)	0.004	1.59 (1.33 ~ 1.91)	<0.001
Middle or high school	1.2 (0.97 ~ 1.48)	0.10	1.55 (1.25 ~ 1.93)	<0.001
College or above	1.15 (0.82 ~ 1.6)	0.42	1.86 (1.35 ~ 2.56)	<0.001
**Rural (vs. urban)**	0.59 (0.47 ~ 0.75)	<0.001	0.29 (0.23 ~ 0.36)	<0.001

OR odds ratio, 95% CI 95% confidence intervals.

In multivariate analyses of physical activity (Table 4), compared to the “Decreasing physical activity” group, those with “Persistently high cognitive function” were more likely to be in the “Persistently low physical activity” group (OR = 2.87, 95% CI: 2.28–3.62), in addition to being more educated (OR = 1.59, 95% CI: 1.03–2.43), older (OR = 1.06, 95% CI: 1.05–1.08), less likely to live in rural areas (OR = 0.47, 95% CI: 0.36–0.63), more likely to have the experience of hospitalization in the past year (OR = 1.55, 95% CI: 1.07–2.24), and more likely to be overweight or obese, with likelihoods of 1.57 (95% CI, 1.13–2.17) and 2.44 (95% CI, 1.59–3.72), respectively. Similarly, compared to the “Decreasing physical activity” group, members of the “Rising physical activity” trajectory group were also more likely to be in the “Persistently high” trajectory group (OR = 1.49, 95% CI: 1.10–2.01), younger (OR = 0.96, 95% CI: 0.94–0.98), and less probability of visual impairment (OR = 0.45, 95% CI: 0.07–0.97, *p* = 0.017).

**Table 4:** Multivariate logistic regression analyses with physical activity trajectory groups.

Variables	Physical activity trajectories *(ref:* Decreasing physical activity*)*			
	Low physical activity		Rising physical activity	
	OR (95%CI)	*p*-value	OR (95%CI)	*p-*value
**Cognitive function (vs. persistently low)**				
Persistently moderate	0.95 (0.78 ~ 1.15)	0.56	1.15 (0.89 ~ 1.48)	0.29
Persistently high	2.87 (2.28 ~ 3.62)	<0.001	1.49 (1.1 ~ 2.01)	0.01
**Age**	1.06 (1.05 ~ 1.08)	<0.001	0.96 (0.94 ~ 0.98)	<0.001
**Educational level (vs. no formal education)**				
Primary school	0.95 (0.78 ~ 1.15)	0.57	1.03 (0.8 ~ 1.31)	0.83
Middle or high school	0.92 (0.73 ~ 1.15)	0.46	0.62 (0.45 ~ 0.86)	0.004
College or above	1.59 (1.03 ~ 2.43)	0.04	1.29 (0.75 ~ 2.22)	0.36
**Rural (vs. urban)**	0.47 (0.36 ~ 0.63)	<0.001	1.26 (0.84 ~ 1.87)	0.26
**BMI (vs. underweight)**				
Normal	1.22 (0.9 ~ 1.65)	0.19	0.96 (0.66 ~ 1.41)	0.85
Overweight	1.57 (1.13 ~ 2.17)	0.01	0.91 (0.6 ~ 1.37)	0.64
Obese	2.44 (1.59 ~ 3.72)	<0.001	1.24 (0.72 ~ 2.11)	0.44
**Visual impairment (vs. No)**	1.14 (0.89 ~ 1.46)	0.30	0.45 (0.07 ~ 0.97)	0.02
**Hospitalization (vs. No)**	1.55(1.07 ~ 2.24)	0.02	1.55(0.99 ~ 2.44)	0.05

OR odds ratio, 95% CI 95% confidence intervals.

### 4.4 Non-response analyses

From the completed CHARLS cohort, 645 participants (4.8%) were excluded due to incomplete baseline data or diagnoses of dementia, Parkinson’s disease, or cognitive impairment. Excluded participants, compared to those included in the current analysis, were more likely to be older, female, have lower levels of education, depressive symptoms, poorer self-reported health, reside in rural areas, have lower incomes, hearing impairment, poorer activity functioning, had lower levels of PA and poorer cognitive functioning at baseline (Supplementary Table S5). An additional 6,841 participants (51.63%) were excluded due to loss to follow-up. Also, they had higher levels of key risk factors, lower levels of physical activity, and poorer cognitive function at baseline (Supplementary Table S6).

### 4.5 Sensitivity analysis

Given that trajectory analyses were more stable over time for participants with three or more observations, we conducted sensitivity analyses for participants who completed all three waves of cognitive functioning and PA. The pattern of cognitive and physical activity trajectories using data from all three waves was similar to the two-wave trajectories (Supplementary Figures S4, S5). As shown in Supplementary Figure S6, the associations between physical activity and cognitive trajectory groups were similar regardless of age (younger: <70 years; older: ≥70 years), sex, depression status, and mobility (all *p*-values > 0.05 for interaction).

## 5 Discussion

In this study, 5,765 older adults were longitudinally followed using the GBDTM, identifying three distinct cognitive and PA trajectories. The study identified demographic and clinical features associated with each trajectory and explored the interactions among these trajectories separately. Most studies have noted that more active participation is associated with better cognitive function (37–39). However, in a recent study from “The China Family Panel Study” in 2010 and 2018, it was found that the positive association between regular physical activity and cognitive functioning was insignificant in 10,691 older adults (40). Differences in the above studies may be due to the level of study design and methodology, which will be further investigated in this study in terms of longitudinal and group heterogeneity.

The cognitive and activity trajectories of different older adults, such as those of different sexes and those in nursing homes, have been studied but with different patterns (24). In terms of cognitive trajectories, a 6-month study of older adults identified three cognitive trajectories in the first 6 months after admission: “always severe,” “always moderate,” and “always intact/mild” (41), in addition, there were findings that categorized cognitive trajectories as stable on average, high and stable, and declining trend, but less than 10% declining (42), in terms of PA trajectories, one study of PA in older men identified three groups of PA trajectories, high, moderate, and low (43), while another study identified four different PA trajectories low, decreasing, increasing and persistently high (44).

Overall, most of the cognitive trajectories of older adults in this study were stable over a 10-year period. 39.9% of Chinese older adults had sustained trajectories of high cognitive functioning, which is consistent with previous studies of hospitalized older adults and older adults in other countries and regions (23). Therefore, stable cognitive trajectories may reflect average cognitive performance and trends in later life. The “persistently high cognitive function” group generally belongs to the group of older adults with more PA, which is a growing trend. Meanwhile, younger, male, higher education level, and living in urban areas are also protective factors. However, we did not observe any significant association between hospitalization and cognitive function. This finding might be attributed to the specific circumstances of hospitalization. Previous research has indicated that unplanned hospitalization may contribute to the long-term impact on cognitive function in older adults, while selective hospitalization may not result in similar outcomes (45).

In addition, 74.2% of older adults had a low and relatively flat trend in PA over 10 years, consistent with findings from a 21-year longitudinal study of women’s health in Australia (23). Thus, low and stable PA may reflect typical activity trajectories in later life for the majority of older adults. Additionally, 12.1% of older adults experienced increased activity over time, and 13.7% experienced a decrease. It is concerning that older adults with declining levels of PA and low physical activity are potentially at an increased risk for cognitive decline and other adverse health outcomes (46). The data indicates that these groups are associated with lower cognitive levels, higher age, unhealthy BMI, lower education levels, rural residence, and visual impairment. The visual impairment associated with retinal aging is of particular concern as it limits mobility and leads to social isolation (47) and depression (48).

Meanwhile, stratified analyses were conducted to further explore the relationship between the two. Previous studies have indicated that the change in the longitudinal phenotypic dimensions of aging is not necessarily linear, as increasing age may affect the outcome of developmental trajectories (13). Cognitive function declines more rapidly after age 70, with the brain’s white matter volume decreasing and ventricular volume increasing rapidly at this age (49). Therefore, we divided the study population into two groups, using 70 years as the threshold. Additionally, the effects of physical activity on cognitive function in older adults may vary by sex and age, since women’s physical mobility and hormone metabolism differ from men’s (28). People of different sex and age have varied preferences and motivations for participating in activities (50). Finally, some studies have indicated that physical activity and cognitive function are more likely to be affected over time due to depression in older Chinese adults (47, 51, 52). However, no statistically significant interactions were found in this study. This may indicate that the GBTM can adequately differentiate between populations with different levels of risk. However, conclusion should be interpreted with caution, as it may also be attributed to the sample.

The strength of the present study lies in its use of long-term and large-scale follow-up data, along with the application of an advanced statistical GBDTM to fit the dual trajectories and explore the multidimensional and dynamic associations between PA and cognition in later life. However, there are several limitations. First, despite using well-validated questionnaires to measure PA and cognition, the TICS scale cannot accurately evaluate individuals’ cognitive status compared to computerized cognitive assessment batteries or clinical dementia screening tools. Although recall bias still exists, we consider it to be a random error. Second, despite conducting multiple robust analyses and adjusting for various demographic characteristics, health behaviors, and health status, we cannot rule out the presence of confounders. Third, the current analyses had a high nonresponse rate. The excluded participants were generally older, less educated at baseline, and had poorer cognitive function and PA. These participants will likely remain in the persistent low cognitive and PA trajectories, based on usual trends, and therefore, are unlikely to significantly impact our findings. Furthermore, 15.6% of subjects followed the trajectories of persistently low physical activity and cognitive function. However, characteristics associated with both sets of trajectories could not be identified. Future studies should incorporate older adult characteristics into the dual trajectory model and conduct further research to examine the risk profile of these individuals to develop tailored care strategies. Finally, due to the observational study design, a causal relationship between the two trajectories could not be established.

## 6 Conclusions and implications

In summary, this study identified three trajectories of PA. While most older adults maintain activity levels in the sustained and low categories, some either improved or declined in PA. Additionally, three trajectories of cognitive functioning were identified, with minimal change over time. The trajectories of PA were closely linked to the trajectories of cognition. Aging is often accompanied by an increased prevalence of degenerative diseases, such as metabolic decline, malnutrition, and visual and auditory impairments (53), which severely affect daily activities and cognition in older adults (54). As recreational physical activity is a non-invasive strategy for combating aging (55), the present study advocates for further research into exercise programs as a therapy to slow the deterioration of physical and cognitive functions. Furthermore, different model structures resulted in distinct classes with contrasting clinical phenotypes, highlighting the importance of identifying subgroups at higher risk of adverse outcomes. This identification is crucial for developing and testing targeted interventions and care pathways. The socio-demographic and clinical characteristics associated with the trajectories can inform clinical staff in categorizing and providing appropriate care.

## Data Availability

The datasets presented in this study can be found in online repositories. The names of the repository/repositories and accession number(s) can be found in the article/Supplementary material.

## Footnotes

^1^http://www.icpsr.umich.edu/icpsrweb/NACDA/studies/36179
