# Supplementary material for: Dual group-based trajectories of physical activity and cognitive function in aged over 55: a nationally representative cohort study
Source: Front Public Health. 2024 Oct 29;12:1450167. doi: 10.3389/fpubh.2024.1450167 (PMC11554534; doi:10.3389/fpubh.2024.1450167)
Supplement: Supplementary file 1 [file Table_1.DOCX]

***Supplement File***

[Figure S1. Sample flowchart 2](#_Toc168340078)

[Figure S.2a. Graphic depictions of group-based trajectory model with two to six groups for cognitive function. 3](#_Toc168340079)

[Table S.2b. Fit statistics for trajectory models for cognitive function. 4](#_Toc168340080)

[Figure S.3a. Graphic depictions of group-based trajectory model with two to six groups for physical activity 5](#_Toc168340081)

[Table S5. Comparison of baseline characteristics between participants included (*n* = 5,765), and excluded due to incomplete baseline data or confirmed diagnosis of dementia and/or Parkinson’s disease or cognitive impairment (*n* = 643) 8](#_Toc168340082)

[Table S6. Comparison of baseline characteristics between participants included (*n* = 5,765) and excluded due to loss to follow-up (*n* = 6,841) 9](#_Toc168340083)

[Supplement Table S7. Association between demographic and clinical characteristics at admission and cognitive impairment trajectory groups. 10](#_Toc168340084)

[Supplement Table S8. Association between demographic and clinical characteristics at admission and cognitive impairment trajectory groups. 11](#_Toc168340085)

[Fig. S4. Trajectories of cognitive scores by increasing age among older adults with completed all three waves cognitive function data. 12](#_Toc168340086)

[Fig. S5. Trajectories of physical activity score by increasing age among older adults with completed all three waves physical activity data. 13](#_Toc168340087)

[Fig.S6. Stratified analysis and interaction for the association of associations of physical activities and cognitive function trajectory group 14](#_Toc168340088)


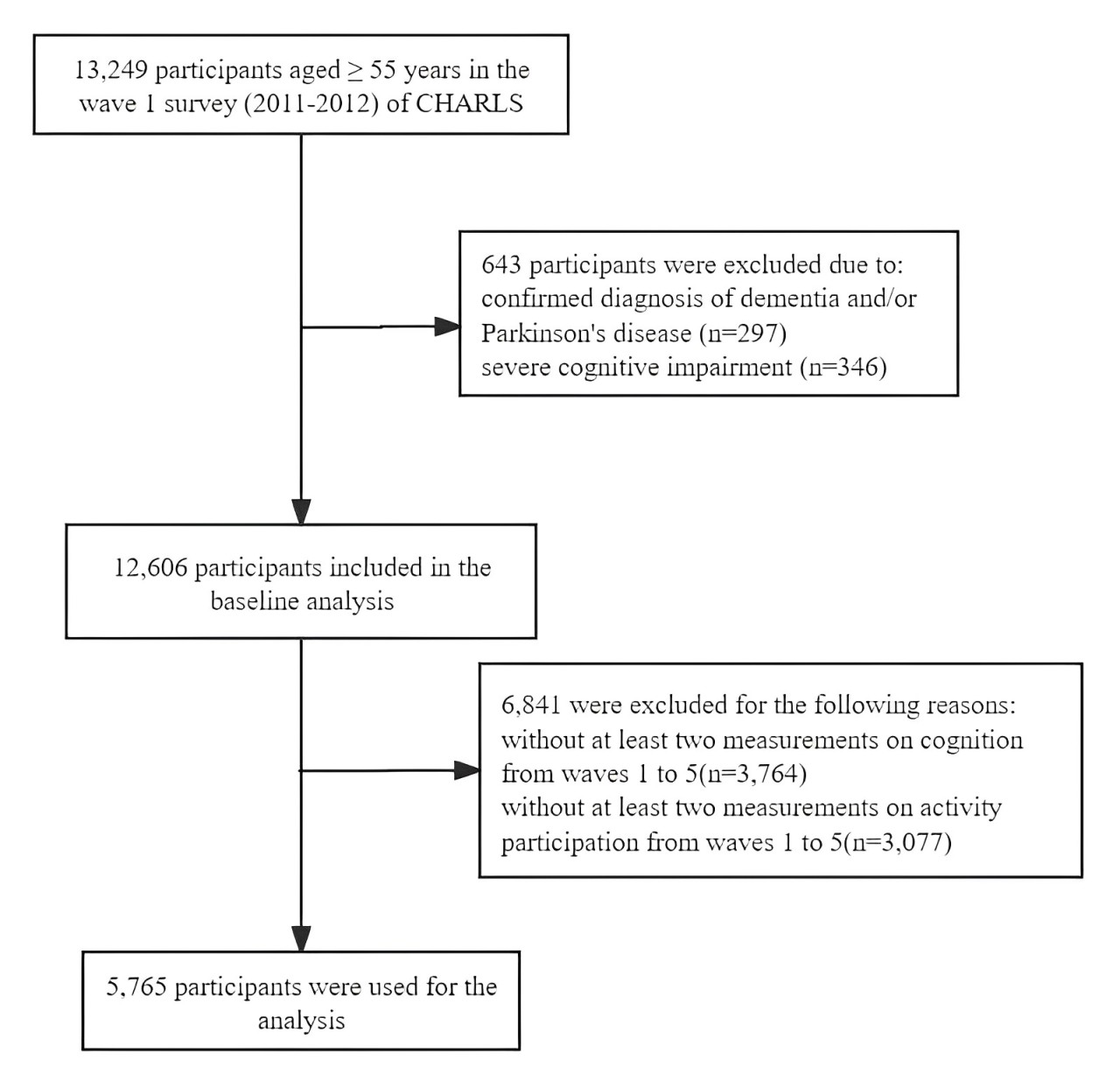


Figure S1. Sample flowchart

Figure S.2a. Graphic depictions of group-based trajectory model with two to six groups for cognitive function.

Table S.2b. Fit statistics for trajectory models for cognitive function.

| **Group** | **Avepp(%)** | **OCC** | **Group membership** | **BIC** | **E_k_** |
| --- | --- | --- | --- | --- | --- |
| **1Group (3)** | 100.00 |  | 100.00 |  | 0.000 |
| **2Group (3 3)** | 92.95-95.46 | 19.3-14.4 | 40.85-59.15 | 3506.22 | 0.810 |
| **3Group (3 3 3)** | **89.54-83.62-90.74** | **30.6-8.5-14.4** | **21.46-37.85-40.69** | **724.26** | **0.740** |
| **4Group (3 3 3 3)** | 85.47-78.08-77.31-84.55 | 39.1-10.1-6.5-15.1 | 12.45-26.89-34.78-25.88 | 179.91 | 0.676 |
| **5Group (3 3 3 3 3)** | 82.59-65.24-65.56-76.30-84.07 | 30.1-12.9-10.4-6.3-16.3 | 14.26-11.45-14.90-35.66-23.73 | 77.59 | 0.652 |
| **6Group (3 3 3 3 3 3)** | 77.23-65.34-62.51-63.55-74.08-82.98 | 45.7-11.3-11.3-14.4-5.9-16.7 | 6.50-15.70-11.40-9.44-35.16-21.80 | 21.01 | 0.635 |

Note: BIC = Bayesian information criterion; AvePP = Fair posterior probability; OCC = Odds of correct classification.

^1^ Defines the shape parameters of the trajectory groups: 0 = intercept only, 1 = linear, 2 = quadratic, 3 = cubic.

^2^ Group AvePP of assignment: Based on the maximum probability assignment rule, each individual will be assigned to a group according to the largest posterior probability. For all the individuals assigned to a certain group, an AvePP will be calculated. For each trajectory group, an AvePP of assignment > 0.7 is indicative of good certainty of group assignments.

^3^ OCC: The numerator is the odds of a correct classification into a certain group based on the model, and the denominator is the correct classification into that group based on random assignment, essentially, OCC = [b/(1-b)]/[a/(1-a)]. For each trajectory group, OCC >= 5 suggests high assignment accuracy.

Figure S.3a. Graphic depictions of group-based trajectory model with two to six groups for physical activity

Table S.3b. Fit statistics for trajectory models for physical activity

| **Group** | **Avepp(%)** | **OCC** | **Group membership** | **BIC** | **E_k_** |
| --- | --- | --- | --- | --- | --- |
| ***1Group (3)*** | 100.00 |  | 100.00 |  | 0.000 |
| ***2Group (3 3)*** | 96.15-89.59 | 6.5-33.1 | 80.40-19.60 | 1509.97 | 0.820 |
| ***3Group (3 3 3)*** | **95.38-81.74-85.34** | **7.1-28.4-42.4** | **75.82-12.16-12.02** | **419.59** | **0.828** |
| ***4Group (3 3 3 3)*** | 78.03-94.53-80.23-80.42 | 37.8-7.2-24.8-57.9 | 8.12-72.42-13.10-6.37 | 233.33 | 0.825 |
| ***5Group (3 3 3 3 3)*** | 78.36-75.19-72.16-80.14-85.22 | 38.9-2.5-15.0-34.4-43.4 | 4.72-70.20-3.14-9.85-12.09 | 94.91 | 0.624 |
| ***6Group (3 3 3 3 3 3)*** | 921.19----- | ----- | 14.75-20.04-34.47-8.51-10.50-11.72 | -24.17 | 0.462 |

Note: BIC = Bayesian information criterion; AvePP = Fair posterior probability; OCC = Odds of correct classification.

1 Defines the shape parameters of the trajectory groups: 0 = intercept only, 1 = linear, 2 = quadratic, 3 = cubic.

2 Group AvePP of assignment: Based on the maximum probability assignment rule, each individual will be assigned to a group according to the largest posterior probability. For all the individuals assigned to a certain group, an AvePP will be calculated. For each trajectory group, an AvePP of assignment > 0.7 is indicative of good certainty of group assignments.

3 OCC: The numerator is the odds of a correct classification into a certain group based on the model, and the denominator is the correct classification into that group based on random assignment, essentially, OCC = [b/(1-b)]/[a/(1-a)]. For each trajectory group, OCC >= 5 suggests high assignment accuracy.

Table S4. Items in the physical activity and cognitive function scale.

| **Variable Wave CHARLS validity CHARLS Variables** | | | |
| --- | --- | --- | --- |
| physical activity | 1-4 | DA051, DA052, DA053, DA054 | DA051（During a usual week, did you do any [….] for at least 10 minutes continuously?）DA052（During a usual week, on how many days did you do [….] for at least 10 minutes?）,DA053（How much time did you usually spend doing [….] on one of those days? ）,DA054（How much time did you usually spend doing [….] on one of those days? ） |
|  | 5 | DA032, DA033, DA034, DA035 | DA032（During a usual week, did you do any [….] for at least 10 minutes continuously?）DA033（During a usual week, on how many days did you do [….] for at least 10 minutes?）,DA034（How much time did you usually spend doing [….] on one of those days? ）,DA035（How much time did you usually spend doing [….] on one of those days? ） |
| cognitive function | 1-4 | DC006/DC007, DC027 | DC006/DC007(Immediately）,DC027（delay） |
|  | 5 | DC012-S1~S10, DC013-S1~S10 | DC014-S1~S10,(Immediately）dc028_s1-10（Delay） |
|  | 1-4 | DC001, DC002, DC003, DC019-DC023, DC025 | DC001(year and month and day) ,DC002(week),DC003(season),DC019-DC023(5，numbers),DC025(draw) |
|  | 5 | DC001, DC002, DC003, DC004, DC005, DCOO7-1~5, DC009 | DC001(year ) ,DC002(season),DC003(day),DC004week),DC005(month),DCOO7-1~5(5，numbers),DC009(draw) |

Table S5. Comparison of baseline characteristics between participants included (*n* = 5,765), and excluded due to incomplete baseline data or confirmed diagnosis of dementia and/or Parkinson’s disease or cognitive impairment (*n* = 643)

| Characteristic | Included (*n* = 5,765) | Excluded (*n* = 643) | *P* for difference |
| --- | --- | --- | --- |
| age, Mean ± *SD* | 62.7 ± 6.2 | 65.9 ± 8.5 | < 0.001 |
| Male sex, *n* (%) | 3117 (54.1) | 3256 (47.6) | < 0.001 |
| Married, *n* (%) | 4497 (87.9) | 474 (73.7) | < 0.001 |
| Educational level, *n* (%) | |  | < 0.001 |
| No formal education | 2379 (46.6) | 476 (74.4) |  |
| Primary school | 1392 (27.2) | 81 (12.7) |  |
| Middle or high school | 885 (17.3) | 57 (8.9) |  |
| College or above | 454 (8.9) | 26 (4.1) |  |
| Self-reported health, *n* (%) | |  | < 0.001 |
| Poor | 1370(27.1) | 262 (70.1) |  |
| Fair | 2568 (50.3) | 239 (37.2) |  |
| Good | 1155 (22.7) | 81 (12.6) |  |
| Depressive symptoms, *n* (%) | 1156 (23.4) | 239 (41.7) | < 0.001 |
| Rural residence, *n* (%) | 4001 (78.3) | 528 (82.1) | 0.024 |
| comorbidity, n (%) | |  | < 0.001 |
| 0 | 1456 (28.5) | 90 (14) |  |
| 1 | 1504 (29.5) | 163 (25.3) |  |
| ≥ 2 | 2146 (42) | 390 (60.7) |  |
| Annual Household Income, *n* (%) | |  | < 0.001 |
| Low | 1168 (33.7) | 221 (44.6) |  |
| Medium | 1128 (32.5) | 166 (33.5) |  |
| High | 1171 (33.8) | 108 (21.8) |  |
| BMI (kg/m^2)^, *n* (%) | |  | < 0.001 |
| Underweight: | 297 (6.9) | 57 (11.3) |  |
| Normal | 2346 (54.3) | 288 (57.1) |  |
| Overweight | 1236 (28.6) | 104 (20.6) |  |
| Obese | 442 (10.2) | 55 (10.9) |  |
| Drinking, *n* (%) | |  | < 0.001 |
| Never | 2898 (56.8) | 399 (62.3) |  |
| Former | 483 (9.5) | 100 (15.6) |  |
| Current | 1721 (33.7) | 141 (22) |  |
| Smoking, *n* (%) | |  | < 0.001 |
| Never | 2832 (56.3) | 398 (65.1) |  |
| Former | 530 (10.5) | 64 (10.5) |  |
| Current | 1664 (33.1) | 149 (24.4) |  |
| Visual Impairment, *n* (%) | 743 (14.6) | 89 (13.9) | 0.651 |
| Hearing Impairment, *n* (%) | 666 (13) | 178 (27.8) | < 0.001 |
| ADL, *n* (%) |  |  | < 0.001 |
| 0 | 4261 (73.9) | 361 (56.1) |  |
| 1 | 456 (7.9) | 92 (14.3) |  |
| ≥2 | 1048 (18.2) | 190 (29.5) |  |
| IADL, *n* (%) |  |  | < 0.001 |
| 0 | 4137 (71.8) | 286 (44.5) |  |
| 1 | 524 (9.1) | 91 (14.2) |  |
| ≥2 | 1104 (19.2) | 266 (41.4) |  |
| cognitive function score, Mean ± *SD* | 12.1 ± 3.1 | 5.6 ± 4.0 | < 0.001 |
| Physical Activity score, Mean ± *SD* | 7301.6 ± 7243.4 | 6141.1 ± 6983.5 | 0.017 |

Note: The differences between participants included and excluded were tested using the t-test or chi-square test.

Table S6. Comparison of baseline characteristics between participants included (*n* = 5,765) and excluded due to loss to follow-up (*n* = 6,841)

| Characteristic | Included (*n* = 5,765) | Loss to follow-up (*n* = 6,841) | *P* for difference |  |
| --- | --- | --- | --- | --- |
| age, Mean ± SD | 62.7 ± 6.2 | 69.1 ± 8.9 | < 0.001 |  |
| Male sex, *n* (%) | 2648 (45.9) | 372 (57.9) | 0.140 |  |
| Married, *n* (%) | 4497 (87.9) | 474 (73.7) | < 0.001 |  |
| Educational level, *n* (%) | |  | < 0.001 |  |
| No formal education | 2379 (46.6) | 3215 (60.4) |  |  |
| Primary school | 1392 (27.2) | 974 (18.3) |  |  |
| Middle or high school | 885 (17.3) | 671 (12.6) |  |  |
| College or above | 454 (8.9) | 460 (8.6) |  |  |
| Self-reported health, *n* (%) | |  | < 0.001 |  |
| Poor | 1380(27.1) | 1789 (33.8) |  |  |
| Fair | 2568 (50.3) | 2454 (46.5) |  |  |
| Good | 1155 (22.7) | 1036 (19.6) |  |  |
| Depressive symptoms, *n* (%) | 1156 (23.4) | 1307 (28.1) | < 0.001 |  |
| Rural residence, *n* (%) | 4001 (78.3) | 3956 (74.3) | 0.024 |  |
| comorbidity, *n* (%) | |  | 0.163 |  |
| 0 | 1456 (28.5) | 1418 (26.9) |  |  |
| 1 | 1504 (29.5) | 1600 (30.3) |  |  |
| ≥ 2 | 2146 (42) | 2263 (42.9) |  |  |
| Annual Household Income, n (%) | |  | 0.003 |  |
| Low | 1168 (33.7) | 1375 (37.5) |  |  |
| Medium | 1128 (32.5) | 1144 (31.2) |  |  |
| High | 1171 (33.8) | 1149 (31.3) |  |  |
| BMI (kg/m^2^), *n* (%) | |  | 0.200 |  |
| Underweight: | 297 (6.9) | 426 (10.9) |  |  |
| Normal | 2346 (54.3) | 2130 (54.4) |  |  |
| Overweight | 1236 (28.6) | 980 (25) |  |  |
| Obese | 442 (10.2) | 381 (9.7) |  |  |
| Drinking, *n* (%) | |  | 0.671 |  |
| Never | 2898 (56.8) | 3303 (62.8) |  |  |
| Former | 483 (9.5) | 543 (10.3) |  |  |
| Current | 1721 (33.7) | 1416 (26.9) |  |  |
| Smoking, *n (*%) | |  | 0.442 |  |
| Never | 2832 (56.3) | 3154 (62.8) |  |  |
| Former | 530 (10.5) | 511 (10.2) |  |  |
| Current | 1664 (33.1) | 1356 (27) |  |  |
| Visual Impairment, *n* (%) | 743 (14.6) | 740 (14) | 0.761 |  |
| Hearing Impairment, *n* (%) | 666 (13) | 1130 (21.4) | 0.051 |  |
| ADL, *n* (%) |  |  | < 0.001 |  |
| 0 | 4261 (73.9) | 3966 (58) |  |  |
| 1 | 456 (7.9) | 535 (7.8) |  |  |
| ≥2 | 1048 (18.2) | 2340 (34.2) |  |  |
| IADL, *n* (%) |  |  | < 0.001 |  |
| 0 | 4261 (73.9) | 3966 (58) |  |  |
| 1 | 456 (7.9) | 535 (7.8) |  |  |
| ≥2 | 1048 (18.2) | 2340 (34.2) |  |  |
| cognitive function score, Mean ± SD | 12.1 ± 3.1 | 11.8 ±3.4 | < 0.001 |  |
| Physical Activity score, Mean ± SD | 7301.6 ± 7243.4 | 5218.6 ± 6401.5 | < 0.001 |  |

Note: The differences between participants included and loss to follow-up were tested using the t-test or chi-square test.

Supplement Table S7. Association between demographic and clinical characteristics at admission and cognitive impairment trajectory groups.

| Variables | | *Cognitive function* trajectories  *(ref: Persistently Low cognitive function)* | | | |
| --- | --- | --- | --- | --- | --- |
|  |  | Persistently Moderate cognitive function |  | Persistently  High cognitive function |  |
|  |  | OR (95%CI) | *P*-value | OR (95%CI) | *P*-value |
| **Physical activity** (ref: Decreasing physical activity) |  | |  |  |  |
| Persistently low | | 0.94 (0.78~1.15) | 0.556 | **2.80 (2.22~ 3.53)** | **<0.001** |
| Rising physical activity | | 1.15 (0.89~1.49) | 0.286 | **1.49 (1.10~ 2.01)** | **0.011** |
| **age** | | **0.97 (0.96~0.98)** | **<0.001** | **0.92 (0.91~ 0.93)** | **<0.001** |
| **Female (vs. Male)** | | **0.80 (0.65~0.98)** | **0.029** | **0.78 (0.63~ 0.96)** | **0.020** |
| Marital status (vs. married) | | 1.02 (0.82~1.27) | 0.852 | 1.00 (0.80~ 1.26) | 0.987 |
| **educational level (vs. No formal education)** | |  |  |  |  |
| Primary school | | 1.30 (1.09~1.54) | 0.003 | 1.58 (1.32~ 1.89) | <0.001 |
| Middle or high school | | 1.19 (0.96~1.48) | 0.113 | 1.52 (1.22~ 1.89) | <0.001 |
| College or above | | 1.15 (0.82~1.62) | 0.412 | 1.76 (1.27~ 2.44) | 0.001 |
| Self-reported health (vs. Poor) | |  |  |  |  |
| Fair | | 1.11 (0.8~1.54) | 0.523 | 0.88 (0.63~ 1.22) | 0.440 |
| Good | | 1.20 (0.83~1.73) | 0.344 | 1.09 (0.75~ 1.58) | 0.666 |
| Depressive symptoms(vs.no) | | 0.87 (0.73~1.04) | 0.13 | 0.86 (0.72~ 1.04) | 0.114 |
| **Rural (vs. Urban)** | | **0.58 (0.45~0.74)** | **<0.001** | **0.30 (0.24~ 0.38)** | **<0.001** |
| comorbidity(vs.0) | |  |  |  |  |
| 1 | | 0.91 (0.75~1.1) | 0.319 | 0.94 (0.77~ 1.15) | 0.571 |
| ≥2 | | 0.91 (0.75~1.1) | 0.338 | 1.04 (0.85~ 1.26) | 0.711 |
| Annual household income (vs. Low) | |  |  |  |  |
| Medium | | 0.9 (0.76~1.06) | 0.217 | 0.86 (0.73~ 1.03) | 0.106 |
| High | | 0.89 (0.74~1.08) | 0.251 | 0.97 (0.80~ 1.18) | 0.788 |
| **BMI (vs. Underweight)** | |  |  |  |  |
| Normal | | 1.21 (0.92~1.59) | 0.169 | 0.98 (0.74~ 1.3) | 0.871 |
| Overweight | | 1.35 (1.00~1.81) | 0.047 | 1.26 (0.93~ 1.7) | 0.139 |
| Obese | | 1.35 (0.95~1.91) | 0.097 | 1.02 (0.71~ 1.47) | 0.899 |
| Drinking (vs. Never) | |  |  |  |  |
| Former | | 1.08 (0.84~1.38) | 0.538 | 0.91 (0.70~ 1.18) | 0.475 |
| Current | | 0.96 (0.80~1.15) | 0.648 | 0.91 (0.75~ 1.09) | 0.313 |
| **Smoking (vs. Never)** | |  |  |  |  |
| Former | | 1.08 (0.83~1.41) | 0.556 | 1.12 (0.86~ 1.46) | 0.401 |
| Current | | 1.30 (1.06~1.58) | 0.011 | 1.12 (0.91~ 1.38) | 0.288 |
| Visual impairment(vs.no) | | 0.97 (0.79~1.2) | 0.798 | 1.07 (0.87~ 1.31) | 0.550 |
| Hearing impairment(vs.no) | | 0.94 (0.77~1.16) | 0.575 | 1.02 (0.83~ 1.26) | 0.843 |
| Hospitalization (vs. No) | | 0.90(0.70~1.15) | 0.392 | 0.85(0.66~1.08) | 0.186 |
| **ADL (vs.no)** | |  |  |  | **0.02** |
| 1 | | 1.01 (0.78~1.32) | 0.914 | 1.05 (0.8~ 1.39) | 0.716 |
| ≥2 | | 1.19 (0.90~1.57) | 0.226 | 1.41 (1.06~ 1.87) | 0.020 |
| IADL (vs.no) | |  |  |  |  |
| 1 | | 1.06 (0.83~1.36) | 0.617 | 0.86 (0.67~ 1.12) | 0.271 |
| ≥2 | | 1.13 (0.86~1.49) | 0.370 | 1.11 (0.84~ 1.48) | 0.462 |

Notes: OR = adjusted odds ratio; CI = confidence interval; Multinomial logistic model with the identified physical activity trajectories as the dependent variable cognitive function trajectories as the main independent variable, adjusting for all other covariates in this table.

Supplement Table S8. Association between demographic and clinical characteristics at admission and cognitive impairment trajectory groups.

| Variables | Physical activity trajectories  *(ref:* Decreasing physical activity*)* | | | | |
| --- | --- | --- | --- | --- | --- |
|  | Persistently Low Physical Activity |  | Rising  physical activity | |  |
|  | OR (95%CI) | *P*-value | OR (95%CI) | *P*-value | |
| **Cognitive function** (vs. Persistently low) |  |  |  | |  |
| Persistently Moderate | 0.94 (0.77~1.14) | 0.52 | 1.16 (0.90~ 1.5) | | 0.251 |
| Persistently High | **2.79 (2.21~3.53)** | **<0.001** | **1.50 (1.10~ 2.03)** | | **0.009** |
| **Age** | 1.06 (1.05~1.08) | <0.001 | 0.90 (0.89~ 0.92) | | <0.001 |
| Female (vs. Male) | 1.03 (0.81~1.31) | 0.818 | 1.25 (0.98~ 1.60) | | 0.068 |
| Marital status (vs. married) | 0.92 (0.72~1.18) | 0.518 | 1.08 (0.83~ 1.41) | | 0.558 |
| **educational level (vs. No formal education)** |  |  |  | |  |
| Primary school | 1.02 (0.83~1.24) | 0.874 | 1.02 (0.84~ 1.24) | | 0.852 |
| Middle or high school | 1.04 (0.82~1.33) | 0.736 | 0.63 (0.48~ 0.84) | | 0.001 |
| College or above | 1.7 (1.1~2.64) | 0.018 | 1.28 (0.73~ 2.23) | | 0.385 |
| Self-reported health (vs. Poor) |  |  |  | |  |
| Fair | 1.03 (0.71~1.49) | 0.89 | 1.2 (0.73~ 1.97) | | 0.477 |
| Good | 1.02 (0.67~1.56) | 0.913 | 1.12 (0.64~ 1.97) | | 0.692 |
| Depressive symptoms (vs. No) | 0.97 (0.79~1.2) | 0.811 | 1.18 (0.91~ 1.54) | | 0.216 |
| **Rural (vs. Urban)** | 0.44 (0.33~0.59) | <0.001 | 1.22 (0.81~ 1.83) | | 0.344 |
| comorbidity(vs.0) |  |  |  | |  |
| 1 | 1.12 (0.9~1.39) | 0.319 | 0.9 (0.68~ 1.2) | | 0.477 |
| ≥2 | 1.08 (0.87~1.35) | 0.467 | 1 (0.75~ 1.32) | | 0.98 |
| Annual household income (vs. Low) |  |  |  | |  |
| Medium | 0.89 (0.73~1.08) | 0.24 | 0.98 (0.76~ 1.25) | | 0.851 |
| High | 0.92 (0.74~1.15) | 0.465 | 0.98 (0.74~ 1.31) | | 0.915 |
| **BMI (vs. Underweight)** |  |  |  | |  |
| Normal | 1.24 (0.91~1.68) | 0.168 | 0.98 (0.67~ 1.43) | | 0.911 |
| Overweight | 1.52 (1.09~2.12) | 0.013 | 0.93 (0.61~ 1.41) | | 0.725 |
| Obese | 2.4 (1.56~3.7) | <0.001 | 1.25 (0.73~ 2.16) | | 0.416 |
| **Drinking (vs. Never**) |  |  |  | |  |
| Former | 0.75 (0.56~0.98) | 0.038 | 0.84 (0.59~ 1.21) | | 0.358 |
| Current | 0.89 (0.73~1.1) | 0.292 | 1.01 (0.77~ 1.32) | | 0.954 |
| Smoking (vs. Never) |  |  |  | |  |
| Former | 1.1 (0.8~1.51) | 0.547 | 0.79 (0.52~ 1.2) | | 0.269 |
| Current | 0.94 (0.75~1.19) | 0.623 | 0.87 (0.64~ 1.16) | | 0.342 |
| **Visual impairment (vs. No)** | 1.13 (0.88~1.45) | 0.341 | 0.43 (0.05~ 0.95) | | 0.024 |
| Hearing impairment (vs. No) | 0.88 (0.7~1.12) | 0.3 | 0.88 (0.65~ 1.19) | | 0.412 |
| Hospitalization (vs. No) | 1.77(1.25~2.49 | 0.001 | 1.48(0.96~2.28) | | 0.007 |
| ADL (vs. No) |  |  |  | |  |
| 1 | 0.77 (0.57~1.03) | 0.081 | 1.01 (0.69~ 1.48) | | 0.954 |
| ≥2 | 1.18 (0.86~1.64) | 0.306 | 1.26 (0.83~ 1.9) | | 0.275 |
| IADL (vs. No) |  |  |  | |  |
| 1 | 1.17 (0.88~1.56) | 0.29 | 0.98 (0.68~ 1.42) | | 0.907 |
| ≥2 | 1.24 (0.91~1.71) | 0.173 | 0.77 (0.51~ 1.16) | | 0.215 |

Notes: Note: OR = adjusted odds ratio; CI = confidence interval; Multinomial logistic model with the identified cognitive impairment trajectories as the dependent variable, physical activity trajectories as the main independent variable, adjusting for all other covariates in this table.

**
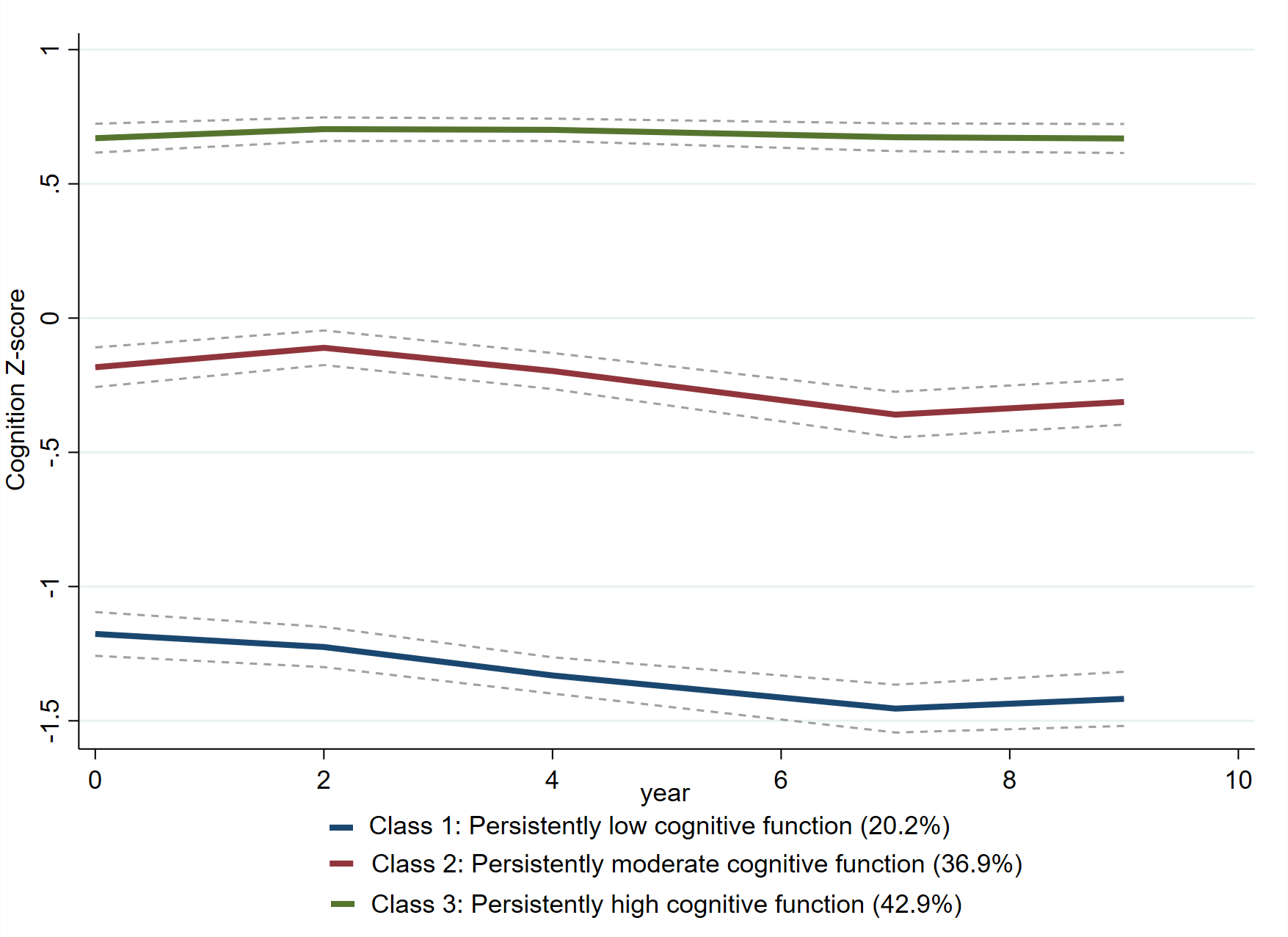
**

Fig. S4. Trajectories of cognitive scores by increasing age among older adults with completed all three waves cognitive function data.


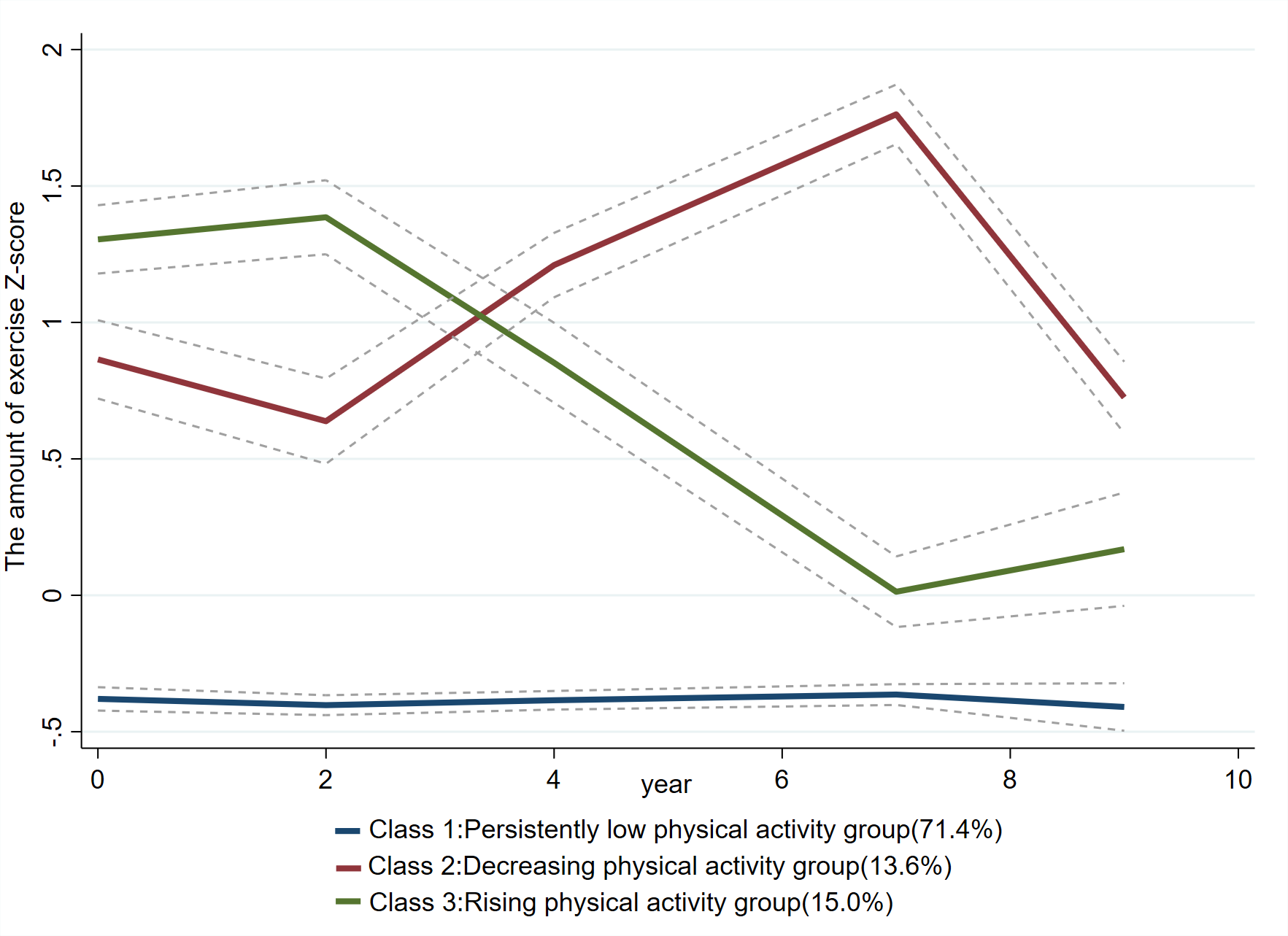


Fig. S5. Trajectories of physical activity score by increasing age among older adults with completed all three waves physical activity data.


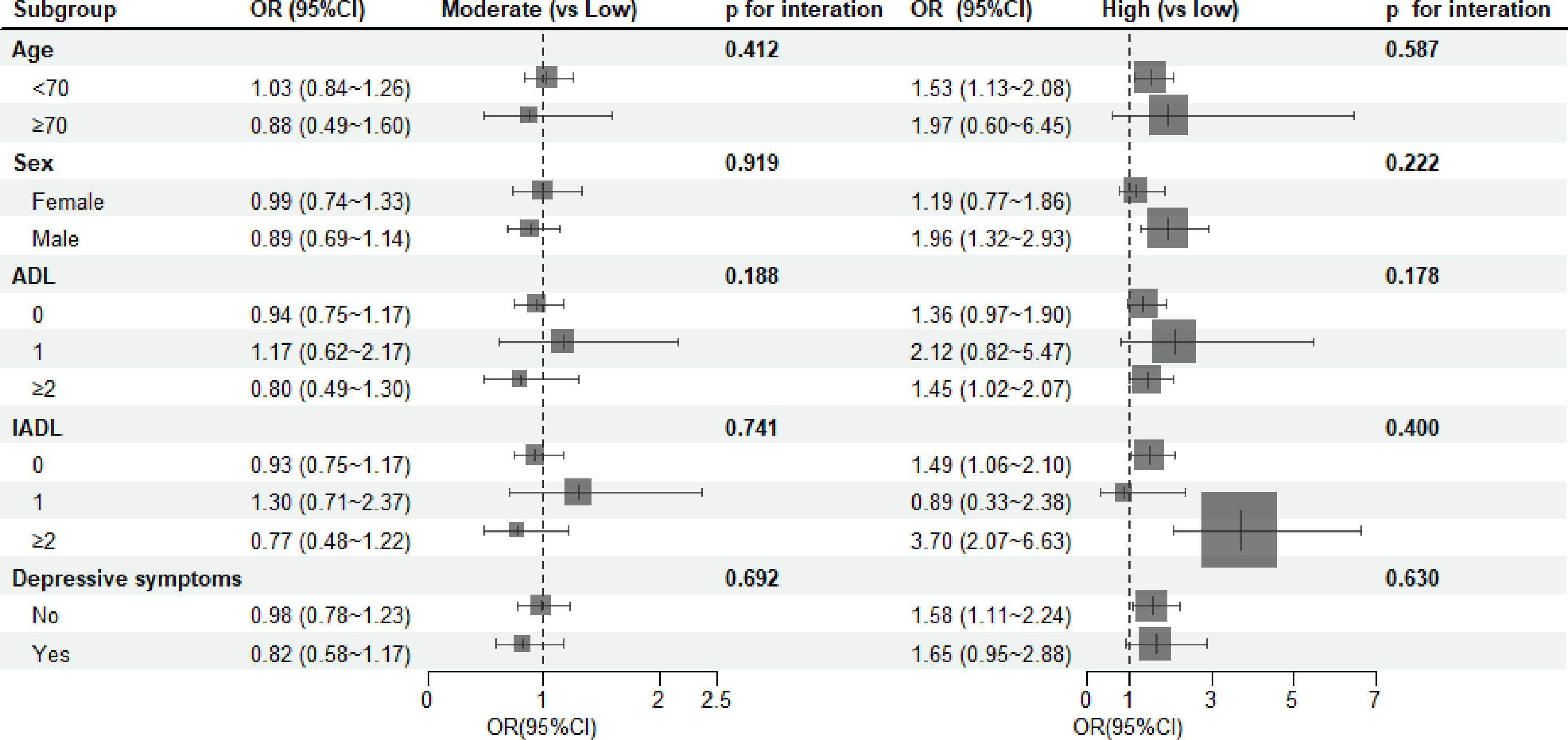


Fig.S6. Stratified analysis and interaction for the association of associations of physical activities and cognitive function trajectory group
